# The germline mutational landscape of genitourinary cancers and its indication for prognosis and risk

**DOI:** 10.1186/s12894-022-01141-1

**Published:** 2022-11-30

**Authors:** Yong Yang, Guoying Zhang, Chen Hu, Wei Luo, Haiyang Jiang, Shaoyou Liu, Hong Yang

**Affiliations:** Department of Urology, The Third Affiliated Hospital of Kunming Medical University, Tumor Hospital of Yunnan Province, Kunming, 650118 Yunnan Province People’s Republic of China

**Keywords:** Genitourinary cancer, Bladder, Kidney, Prostate, Germline, Mutation, Hereditary, Prognosis

## Abstract

**Background:**

Germline mutations represent a high risk of hereditary cancers in population. The landscape and characteristics of germline mutations in genitourinary cancer are largely unknown, and their correlation with patient prognosis has not been defined.

**Methods:**

Variant data and relevant clinical data of 10,389 cancer patients in The Cancer Genome Atlas (TCGA) database was downloaded. The subset of data of 206 genitourinary cancer patients containing bladder urothelial carcinoma (BLCA), kidney chromophobe carcinoma (KICH), kidney renal clear cell carcinoma (KIRC), kidney renal papillary cell carcinoma (KIRP) and prostate adenocarcinoma (PRAD) cancer with germline mutation information was filtered for further analysis. Variants were classified into pathogenic, likely pathogenic and non-pathogenic categories based on American College of Medical Genetics and Genomics (ACMG) guidelines. Genome Aggregation Database (gnomAD) database was used to assist risk analysis.

**Results:**

There were 48, 7, 44, 45 and 62 patients with germline mutations identified in BLCA, KICH, KIRC, KIRP and PRAD, respectively. Pathogenic germline mutations from 26 genes and likely pathogenic mutations from 33 genes were revealed. *GJB2*, *MET*, *MUTYH* and *VHL* mutations ranked top in kidney cancers, and *ATM* and *CHEK2* mutations ranked top for bladder cancer, while *ATM* and *BRCA1* mutations ranked top for prostate cancer. Frameshift, stop gained and missense mutations were the predominant mutation types. BLCA exhibited the highest ratio of stop gained mutations (22/48 = 45.8%). No difference in patient age was found among pathogenic, likely pathogenic and non-pathogenic groups for all cancer types. The number of male patients far overweight female patients whether PRAD was included (*P* = 0) or excluded (*P* < 0.001). Patients with pathogenic or likely pathogenic germline mutations exhibited significantly worse overall survival rate than the non-pathogenic group for all genitourinary cancers. More important, analyses assisted by gnomAD database revealed that pathogenic or likely pathogenic germline mutations significantly increased the risk for genitourinary cancer in population, with the odds ratio at 14.88 (95%CI 11.80–18.77) and 33.18 (95%CI 24.90–44.20), respectively.

**Conclusions:**

The germline mutational status for genitourinary cancers has been comprehensively characterized. Pathogenic and likely pathogenic germline mutations increased the risk and indicated poor prognosis of genitourinary cancers.

## Introduction

Genitourinary cancers include bladder carcinoma (BLCA), kidney chromophobe carcinoma (KICH), kidney renal clear cell carcinoma (KIRC), kidney renal papillary cell carcinoma (KRIP) and prostate adenocarcinoma (PRAD). The age-standardized rate (ASR) of bladder cancer, renal cell cancers and prostate cancer was reported to be 9.0 [1] 15.80 [2] and 29.3 [3] per 100,000 people in men, respectively, and 2.2 [1] and 7.56 [2] per 100,000 people in women, respectively. People in North American and Europe generally exhibited higher incidence than people in Asia and Africa [1–3].

Approximately 10–15% of cancer patients belong to hereditary cancer, characterized by strong hereditary background known as pathogenic germline mutations [4–6]. These patients generally inherit pathogenic mutations from their parents and have high risk of cancer than people without germline mutations. Many of them show cancer phenotypes at earlier stage of life than average risk population who may have sporadic cancers at elder ages. They may pass germline mutations to the next generation, thus increasing the cancer risk of their children. Genitourinary cancer with germline mutations represents a specific type of cancers with strong hereditary background. Reports on individual genitourinary cancer types showed strong link between the onset of cancer with pathogenic germline mutations, including prostate cancer [7] and urothelial cancer [8]. Genes involved in germline alterations of genitourinary cancer included those in DNA damage and repair (DDR) pathways, such as *ATM*, *BRCA1*, *BRCA2*, *MLH1* and *MSH2* [9, 10].

Although there are some reports available on germline mutations in certain types of genitourinary cancers, the full profile and characteristics of germline alterations in all genitourinary cancers have not been investigated in detail. It is also unclear on the correlation between germline alterations and patient phenotypes and prognosis. The risk caused by germline alterations in genitourinary cancers has not been quantified. Here we performed a database study and characterized the profile of germline mutations and their links with patient phenotypes, risk and prognosis for five individual genitourinary cancers. We aimed to provide useful information for future prevention, early intervention and treatment of genitourinary cancer patients with germline alterations.

## Methods and materials

Germline variants and relevant clinical data of 10,389 cancer patients corresponding to 33 cancer types were downloaded from the TCGA database (generated by Huang et al.[11]) as the input dataset (https://www.sciencedirect.com/science/article/pii/S0092867418303635). A subset of 206 genitourinary cancer patients containing BLCA, KICH, KIRC, KIRP and PRAD were filtered for further analysis. All variants were classified into pathogenic, likely pathogenic and non-pathogenic subgroups based on American College of Medical Genetics and Genomics (ACMG) guidelines [12]. A summary of the patient demographic and clinicopathological information is presented as Table 1.

**Table 1:** Demographic and clinicopathological information for subjects included in this study

Factors	Categories	Pathogenecity, number (%)			*P*
		Pathogenic	Likely pathogenic	Non-pathogenic	
*Gender*					
	Female	21 (35.6)	10 (19.2)	15 (15.8)	0.013
	Male	38 (64.4)	42 (80.8)	80 (84.2)	
*Age*					
	< 40	2 (3.4)	3 (5.8)	3 (3.2)	0.963
	40–49	6 (10.2)	6 (11.5)	9 (9.5)	
	50–59	19 (32.2)	17 (32.7)	25 (26.3)	
	60–69	17 (28.8)	12 (23.1)	33 (34.7)	
	70–79	12 (20.3)	10 (19.2)	20 (21.0)	
	> = 80	3 (5.1)	4 (7.7)	5 (5.3)	
*Stage*					
	I	7 (11.9)	5 (9.6)	10 (10.5)	0.685
	II	0 (0.0)	2 (3.9)	2 (2.1)	
	III	4 (6.8)	1 (1.9)	2 (2.1)	
	IV	0 (0.0)	1 (1.9)	2 (2.1)	
	Not specified	48 (81.3)	43 (82.7)	79 (83.2)	
*Race*					
	Asian	1 (1.7)	2 (3.8)	4 (4.2)	0.688
	African American	8 (13.6)	8 (15.4)	7 (7.4)	
	White	38 (64.4)	31 (59.6)	59 (62.1)	
	Not specified	12 (20.3)	11 (21.2)	25 (26.3)	
*Cancer*					
	BLCA	17 (28.8)	13 (25.0)	18 (18.9)	0.736
	KICH	2 ( 3.4)	2 ( 3.8)	3 (3.2)	
	KIRC	14 (23.7)	8 (15.4)	22 (23.2)	
	KIRP	12 (20.4)	14 (27.0)	19 (20.0)	
	PRAD	14 (23.7)	15 (28.8)	33 (34.7)	
*Ethnicity*					
	African	11 (18.6)	10 (19.2)	13 (13.7)	0.774
	American	1 (1.7)	2 (3.8)	2 (2.1)	
	Asian	1 (1.7)	2 (3.8)	4 (4.2)	
	European	46 (78.0)	37 (71.3)	76 (80.0)	
	Mix	0 (0.0)	1 (1.9)	0 (0.0)	
	Total	59	52	95	

Information on variants from different variant categories was collected, and was grouped by gene names or cancer types, and was ranked in descending order to identify the high-frequency variants. The distribution of mutational categories and pathogenicity was plotted by R software. Variants in representative genes were displayed as lollipop plots by the R software. Variants located outside the exon regions were not displayed in plots. Wilcoxon tests were performed to compare the age among groups with different pathogenicity. Chi-square test or fisher exact test was used to determine the significance among rates or percentage. The Kaplan–Meier analysis and log-rank test were used to analyze and compare the overall survival rate among different groups. Variant frequency data in population from the gnomAD database was used to calculate the risk of germline mutations. The odds ratio (OR) values were calculated based on the variant frequency from the database and in this study. The significance of OR values was assessed by Fisher’s exact test and *P* values were adjusted by the Benjamini and Hochberg (BH) method. *P* < 0.05 was regarded as statistical significant difference.

## Results

### The landscape and characteristics of germline mutations in genitourinary cancer

The mutation data of a total of 206 patients with genitourinary cancers were collected. The demographic and clinicopathological information of all patients is summarized in Table 1. The number of male patients far overweight that of the female patients (*P* = 0.013). The race of patients was mainly white with cancer stage information invalid for the majority of patients. No difference in age was found among the pathogenic, likely pathogenic and non-pathogenic groups. Five cancers were involved in this study, including BLCA, KICH, KIRC, KIRP and PRAD.

**Fig. 1:**
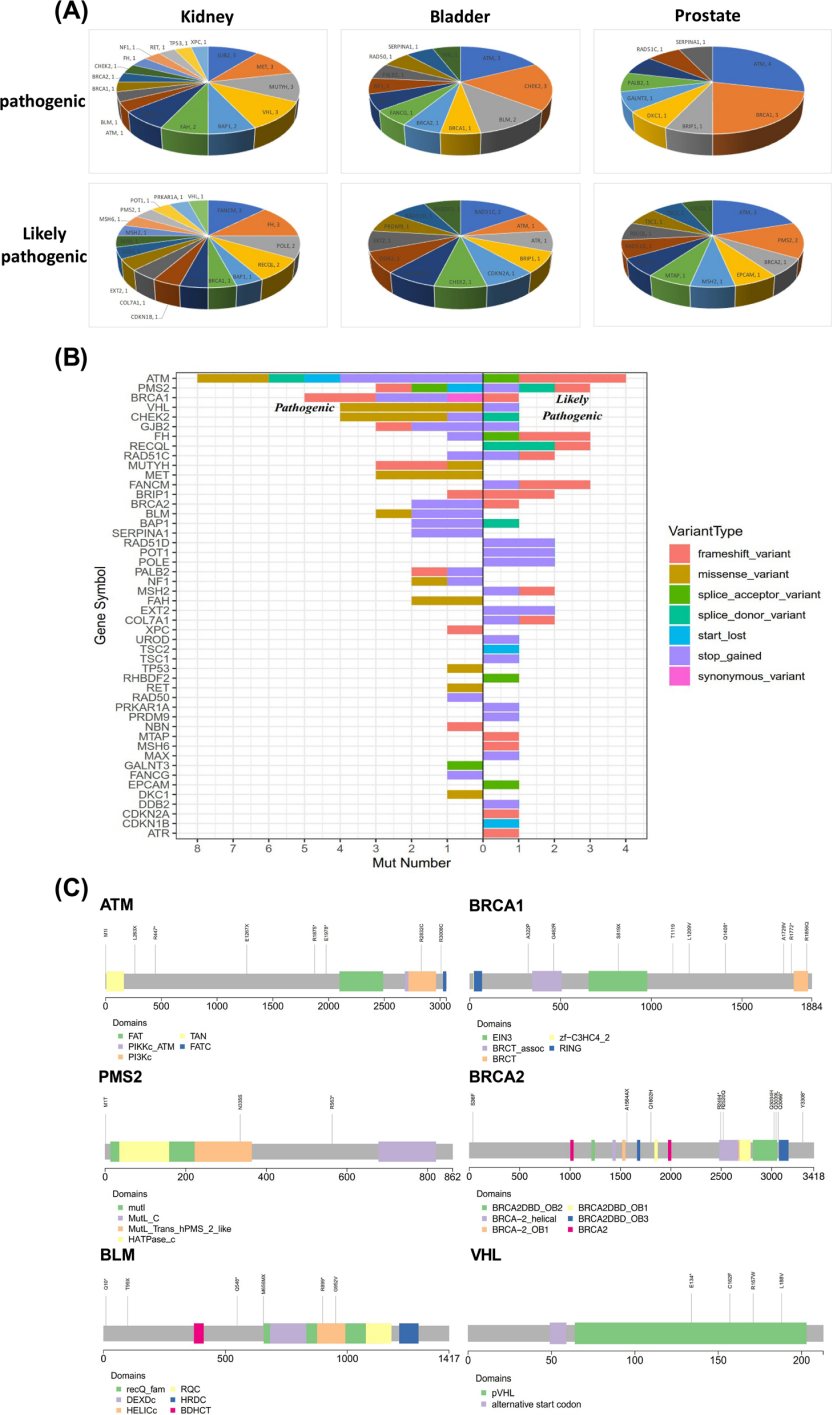
The germline mutational status and distribution for kidney, bladder and prostate cancer. **A** the number of pathogenic and likely pathogenic mutations for each individual cancers; **B** the mutation types for genes with pathogenic or likely pathogenic mutations in all cancers. **C** schemes show the distribution of individual germline mutations for representative genes. Please be noted that variants located outside the exon region, mainly splicing variants, were not plotted in the schemes

The distribution of germline mutations in genitourinary cancers was investigated first. All germline mutations were divided into pathogenic, likely pathogenic and non-pathogenic based on ACMG guidelines. The numbers and types of pathogenic and likely pathogenic mutations for kidney, bladder and prostate cancers are shown in Fig. 1A. It can be observed that for pathogenic mutations, *GJB2*, *MET*, *MUTYH* and *VHL* ranked top in kidney cancer, and *ATM* and *CHEK2* ranked top for bladder cancer, while *ATM* and *BRCA1* ranked top for prostate cancers. For likely pathogenic mutations, *FANCM* and *FH* ranked top for kidney cancer, *RAD51C* ranked top for bladder cancer, and *ATM* and *PMS2* ranked top for prostate cancer. Pathogenic germline mutations from 26 genes and likely pathogenic mutations from 33 genes are shown in Fig. 1B. *ATM* germline mutations ranked the first in number in all pathogenic mutations, followed by *BRCA1*, *CHEK2*, *VHL* and *BLM*. *ATM* also ranked the highest in likely pathogenic mutations, followed by *FANCM*, *FH* and *PMS2* (Fig. 1B). The distribution of mutation types for all involved genes is shown in Fig. 1B, grouped by mutation pathogenicity (pathogenic or likely pathogenic). The main mutation types included frameshift, stop gained and missense mutations, while other less frequent types, such as splicing and start lost mutations were also present (Fig. 1B). The mutational distribution of representative genes is shown in Fig. 1C, including *ATM*, *BRCA1*, *BRCA2*, *PMS2*, *BLM* and *VHL*. The mutational distribution appeared to be random and no obvious hotspot mutations were found.

**Fig. 2:**
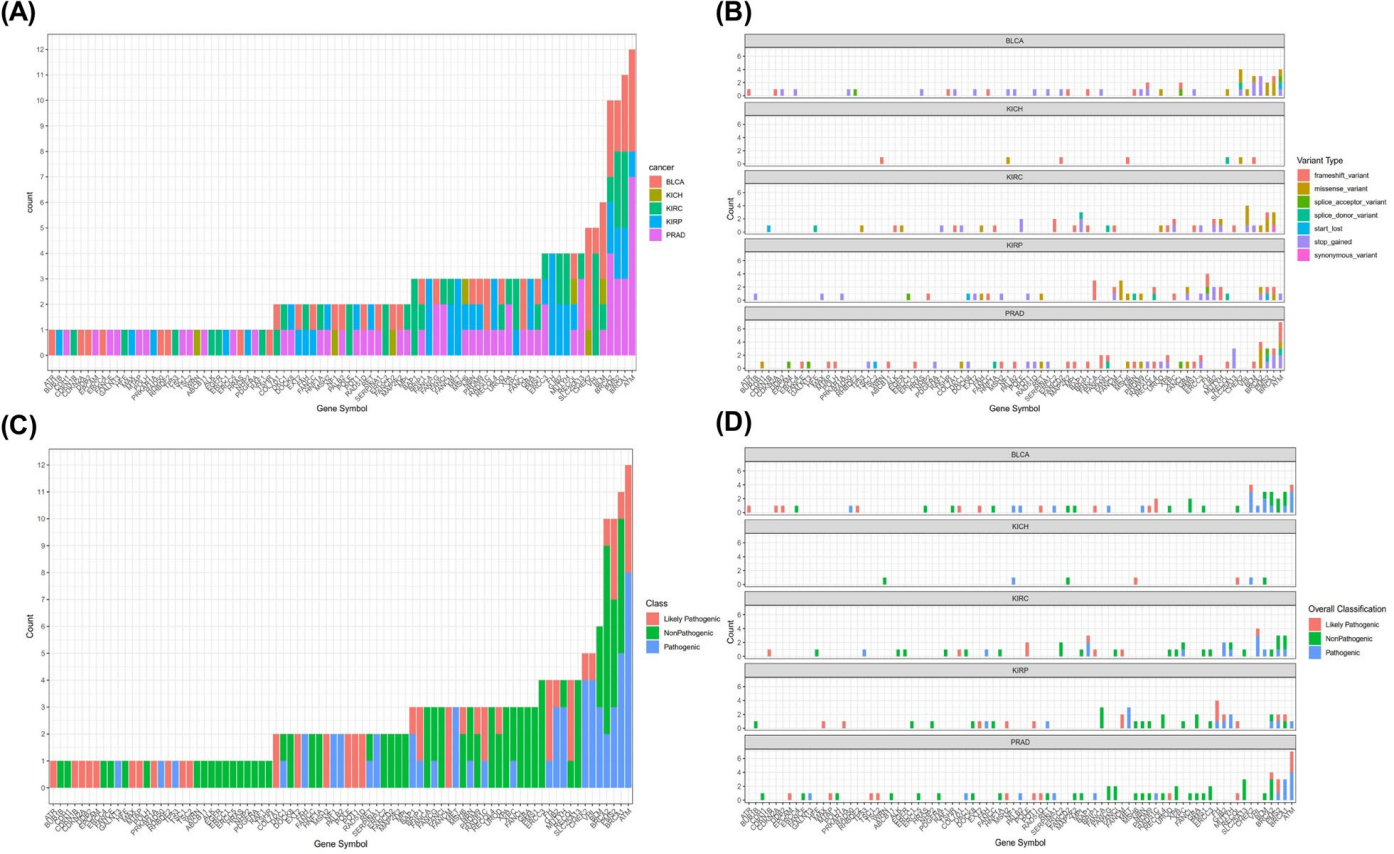
Germline mutational status in five genitourinary cancers. **A** the distribution of germline mutations for each gene in BLCA, KICH, KIRC, KIRP and PRAD; **B** landscape of mutation types for each individual cancer types; **C** distribution of mutation pathogenicity across all involved genes; **D** landscape of mutation pathogenicity for each individual cancer types

The mutational distribution of the five types of genitourinary cancers is shown in Fig. 2A. There were 48, 7, 44, 45 and 62 patients with mutations found in BLCA, KICH, KIRC, KRIP and PRAD, respectively (Fig. 2A, Table 2). *ATM*, *BRCA1*, *PMS2* and *BRCA2* were among those with highest number of mutations. The distribution of mutation types for each cancer type is shown in Fig. 2B. BLCA had higher ratio of stop gained mutations (22/48 = 45.8%) compared with other four types of cancers (44/158 = 27.8%) (*P* = 0.019), suggesting a preference of the specific type of germline mutation for BLCA (Table 2). The status of pathogenicity for each gene is shown in Fig. 2C. Pathogenic mutations were mainly found in high frequency mutated genes while non-pathogenic mutations were also present in large majority of genes. This can also be observed in Fig. 2D, in which the status of pathogenicity for each cancer type is shown. No difference in the distribution of pathogenicity status across the five cancer types was found (*P* = 0.74) (Table 2).

**Table 2:** The number of patients for each cancer type grouped by sex, variant type and pathogenicity

Factors	Categories	Cancer Types					*P* values
		BLCA	KICH	KIRC	KIRP	PRAD	
*Sex (%)*							
	Female	10 (20.8)	2 (28.6)	25 (56.8)	9 (20.0)	0 ( 0.0)	*P* = 0.00039(excl PRAD)
	Male	38 (79.2)	5 (71.4)	19 (43.2)	36 (80.0)	62 (100.0)	*P* < 0.001(incl PRAD)
*Variant_Type (%)*							
	stop_gained	22 (45.8)	0 ( 0.0)	12 (27.3)	14 (31.1)	18 (29.0)	*P* = 0.37
	frameshift_variant	10 (20.8)	4 (57.1)	14 (31.8)	14 (31.1)	25 (40.3)	
	missense_variant	11 (22.9)	2 (28.6)	13 (29.5)	10 (22.2)	10 (16.1)	
	Others	5 (10.5)	1 (14.3)	5 (11.4)	7 (15.6)	9 (14.5)	
*Pathogenicity (%)*							
	Likely Pathogenic	13 (27.1)	2 (28.6)	8 (18.2)	14 (31.1)	15 (24.2)	*P* = 0.74
	NonPathogenic	18 (37.5)	3 (42.9)	22 (50.0)	19 (42.2)	33 (53.2)	
	Pathogenic	17 (35.4)	2 (28.6)	14 (31.8)	12 (26.7)	14 (22.6)	
Number of patients		48	7	44	45	62	

### The influences of germline mutations on patient phenotypes and prognosis

**Fig. 3:**
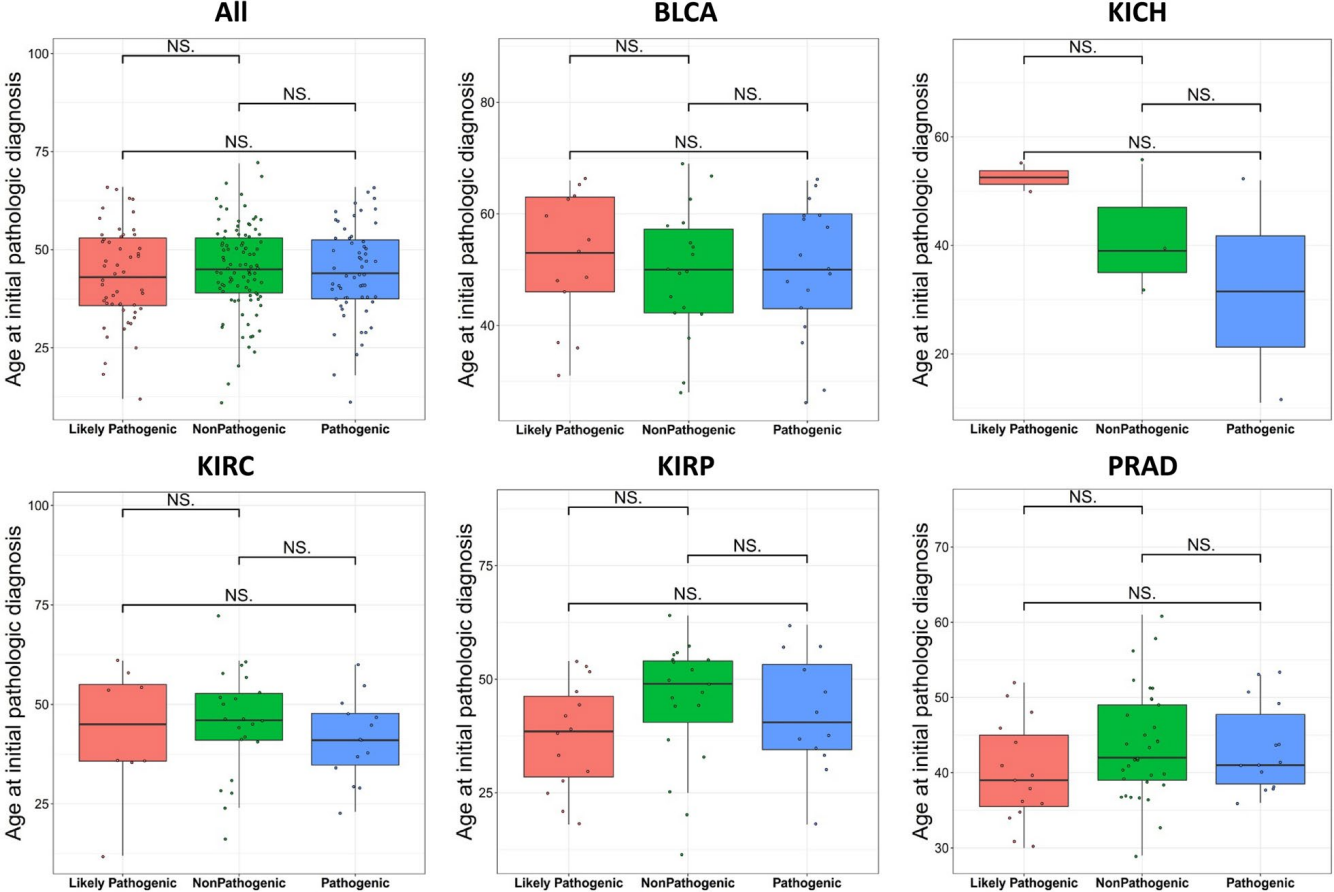
Comparison of cancer onset age among pathogenic, likely pathogenic and non-pathogenic groups. No difference (NS.) was found among the three groups for all cancers and each individual cancers

We further investigated the correlation between germline mutations and patient age and sex. It can be clearly seen from Fig. 3 that no difference in patient age was found among pathogenic, likely pathogenic and non-pathogenic groups for all genitourinary cancer patients or five individual cancer types (Fig. 3, as labeled), suggesting that the germline mutations had no influence on cancer onset age. The number of male patients far overweight that of the female patients (male:female = 160:46) for all patients across the five cancer types (*P* < 0.001), and the observation was also true even if PRAD patients were excluded (male:female = 98:46) (*P* = 0.00039) (Table 2). Interestingly, the number of female overweight that of the male in KIRC (female:male = 25:19), exhibiting significant difference to other genitourinary cancer (*P* < 0.001), even if PRAD was excluded (*P* = 0.00002) (Table 2).

**Fig. 4:**
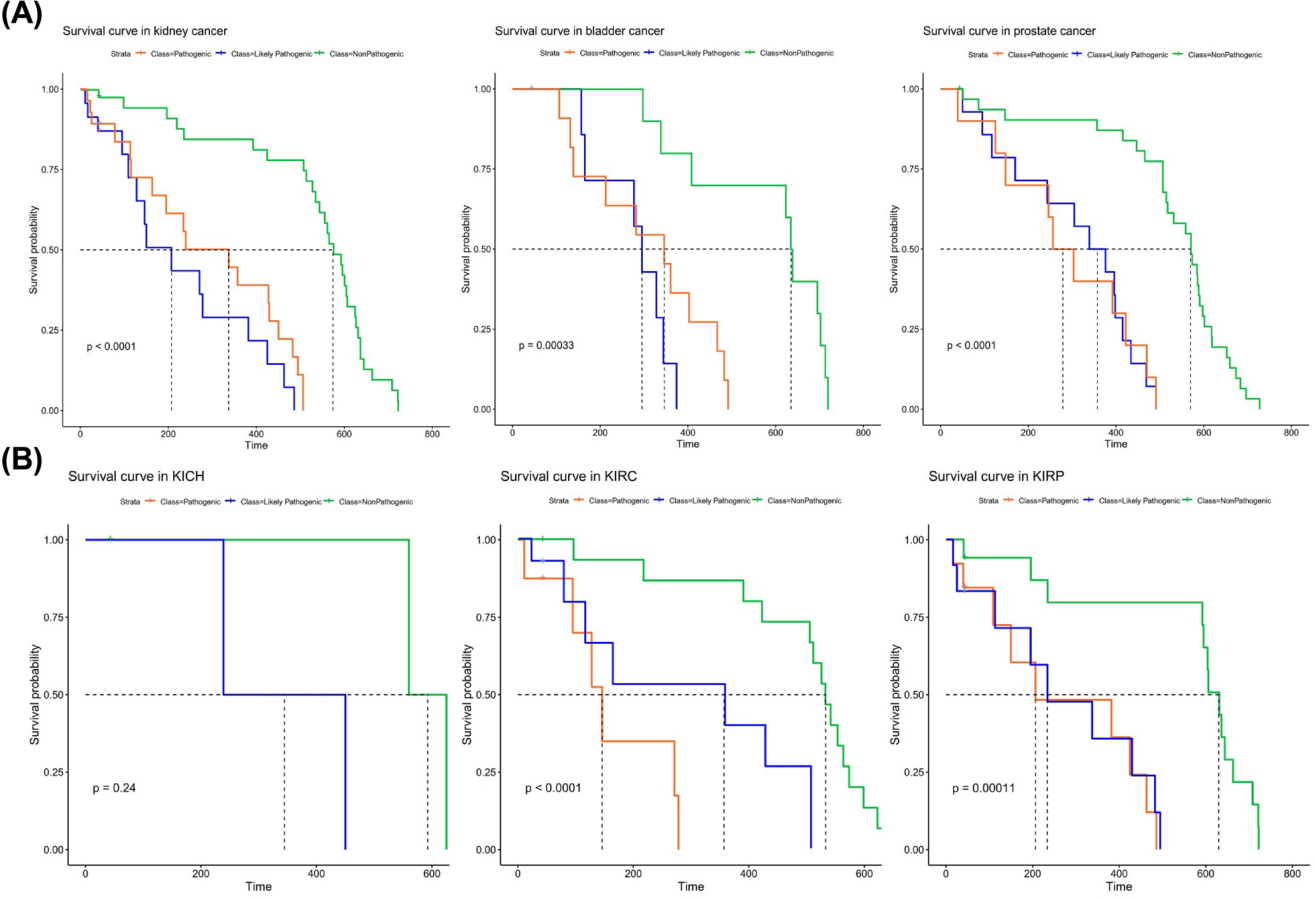
The prognosis of kidney, bladder and prostate cancer patients was affected by the pathogenicity of germline mutations. Significantly better overall survival was observed in non-pathogenic group (green lines) compared with pathogenic (orange lines) or likely pathogenic group (blue lines) in kidney, bladder and prostate cancer (**A**), and in subtypes of kidney cancer, including KIRC and KIRP (**B**). *P* values for each cancer are labeled. The *P* values between any two subgroups are listed below: kidney cancer (*P* vs. LP = 0.18; *P* vs. Non-*P* < 0.001; LP vs. Non-*P* < 0.001); bladder cancer (*P* vs. LP = 0.2024; *P* vs. Non-*P* = 0.0015; LP vs. Non-*P* = 0.0011); prostate cancer (*P* vs. LP = 0.81; *P* vs. Non-*P* < 0.001; LP vs. Non-*P* < 0.001); KICH (*P* vs. LP = 1.00; *P* vs. Non-*P* = 0.27; LP vs. Non-*P* = 1.00); KIRC (*P* vs. LP = 0.1108; *P* vs. Non-*P* = 0.0014; LP vs. Non-*P* < 0.001); KIRP (*P* vs. LP = 0.7304; *P* vs Non-*P* = 0.0004; LP vs. Non-*P* = 0.0004)

The influence of germline mutations on patient overall survival was investigated in detail (Fig. 4). Kidney, bladder and prostate cancer patients with no pathogenic germline mutations (non-pathogenic group, green lines) exhibited significantly better overall survival than those with pathogenic (orange lines) or likely pathogenic mutations (blue lines) (*P* values are shown as indicated; Fig. 4A). Specifically, we found *P* < 0.001 when pathogenic or likely pathogenic group was compared with non-pathogenic group in kidney cancer and prostate cancer, and *P* < 0.01 was found in the above comparisons in bladder cancer. For subtypes of kidney cancers, patients with non-pathogenic mutations exhibited significantly better overall survival rate than those with pathogenic or likely pathogenic mutations in KIRC and KIRP (*P* values are shown as indicated, Fig. 4B). Specifically, we found *P* < 0.01 when pathogenic or likely pathogenic group was compared with non-pathogenic group in both KIRC and KIRP. However, no significant difference was found between non-pathogenic and likely pathogenic group in KICH (*P* > 0.05), possibly due to the limited number of patients in this cancer type. No significant difference in overall survival rate between pathogenic or likely pathogenic groups was found in all cancers (*P* > 0.05).

### The impact of germline mutations on genitourinary cancer risk

To investigate the risk for genitourinary cancers in individuals carrying pathogenic or likely pathogenic germline mutations, the mutation prevalence of all germline mutations in general population was determined by searching the gnomAD (Table 3 for pathogenic and Table 4 for likely pathogenic mutations). By comparing the germline mutation frequency found in this study with the variant prevalence in general population, the overall OR was calculated for germline mutations in this study to avoid the bias from individual mutations. Table 3 presents a summary of demographic information, mutational status and OR for those with pathogenic mutations. The OR for individual mutations in each type of cancer is listed. The overall OR value of all pathogenic mutations was 14.88 (95% CI 11.80–18.77), when compared with the general population. Similarly, Table 4 summarizes the demographic information, mutational status and OR for those with likely pathogenic mutations, and the overall OR value was 33.18 (95% CI 24.90–44.20) when compared with the general population. The significance of each OR was assessed by the adjusted *P* value and was presented in both tables. These analyses suggested that the pathogenic and likely pathogenic germline mutations were significant risk factors for genitourinary cancer.

**Table 3:** Summary of patient and mutational status and OR for patients with pathogenic germline mutations in this study

Number	Age	Gender	Cancer type	Race	Gene	Protein change	Annotation	Allele freqency in population	OR	95% CI	Adjusted P
1	28	MALE	BLCA	WHITE	ATM	p.E1978*	Pathogenic	0.00004379(11/251182)	111.38	24.61 to 504.09	1.33405E−23
2	26	FEMALE	BLCA	BLACK OR AFRICAN AMERICAN	ATM	p.M1I	Pathogenic	0.00000398(1/251388)	611.65	38.19 to 9795.74	9.95953E−18
3	46	FEMALE	BLCA	WHITE	ATM	p.R2832C	Pathogenic	0.00003184(8/251268)	76.42	9.54 to 612.38	7.13507E−05
4	60	FEMALE	BLCA	WHITE	BLM	p.G952V	Pathogenic	0.00000398(1/251446)	611.79	38.20 to 9798.00	9.95953E−18
5	37	MALE	BLCA	WHITE	BLM	p.R899*	Pathogenic	0.00006365(18/282794)	38.22	5.09 to 287.00	0.004715125
6	60	MALE	BLCA	WHITE	BRCA1	p.S819X	Pathogenic	0.00001196(3/250740)	203.36	21.11 to 1959.09	1.81953E−09
7	49	MALE	BLCA	ASIAN	BRCA2	p.R2494*	Pathogenic	0.00003182(8/251440)	76.47	9.54 to 612.80	7.13507E−05
8	50	FEMALE	BLCA	WHITE	CHEK2	p.S471F	Pathogenic	0.00046420(131/282204)	15.79	5.01 to 49.81	2.63664E−07
9	58	FEMALE	BLCA	WHITE	CHEK2	p.S471F	Pathogenic	0.00046420(131/282204)	15.79	5.01 to 49.81	2.63664E−07
10	59	MALE	BLCA	WHITE	CHEK2	p.W93*	Pathogenic	0.00002388(6/251270)	101.89	12.24 to 848.24	5.9444E−06
11	43	MALE	BLCA	WHITE	FANCG	p.E105*	Pathogenic	0.00001425(4/280654)	170.71	19.04 to 1530.66	1.32534E−08
12	66	MALE	BLCA	WHITE	NBN	p.KQ233-234X	Pathogenic	0.00001999(5/250094)	121.70	14.19 to 1043.98	1.08043E−06
13	63	FEMALE	BLCA	WHITE	NF1	p.R1362*	Pathogenic	0.00000398(1/251344)	611.54	38.18 to 9794.02	9.95953E−18
14	53	FEMALE	BLCA	WHITE	PALB2	p.R753*	Pathogenic	0.00002386(6/251486)	101.98	12.25 to 848.97	5.9444E−06
15	65	MALE	BLCA	WHITE	RAD50	p.R1077*	Pathogenic	0.00002123(6/282600)	114.60	13.77 to 954.00	1.54259E−06
16	40	MALE	BLCA	WHITE	SERPINA1	p.Y184*	Pathogenic	0.00017670(50/282896)	27.59	6.69 to 113.79	3.14159E−07
17	48	MALE	BLCA	WHITE	VHL	p.L188V	Pathogenic	0.00002121(6/282824)	114.69	13.78 to 954.76	1.54259E−06
18	52	MALE	KICH	WHITE	CHEK2	p.S471F	Pathogenic	0.00046420(131/282204)	102.53	31.80 to 330.64	2.62808E−43
19	11	FEMALE	KICH	WHITE	NF1	p.K1444E	Pathogenic	NA	NA	NA	NA
20	37	FEMALE	KIRC	WHITE	BAP1	p.Q684*	Pathogenic	0.00000398(1/251330)	1305.61	118.14 to 14,429.10	1.3624E−106
21	41	FEMALE	KIRC	BLACK OR AFRICAN AMERICAN	BAP1	p.Q684*	Pathogenic	0.00000398(1/251330)	1305.61	118.14 to 14,429.10	1.3624E−106
22	60	FEMALE	KIRC	WHITE	BLM	p.Q548*	Pathogenic	0.00016650(46/276228)	15.55	2.14 to 113.07	0.091089752
23	48	FEMALE	KIRC	WHITE	BRCA1	p.T1119	Pathogenic	0.00000400(1/250270)	648.37	40.48 to 10,384.92	1.16019E−18
24	55	MALE	KIRC	WHITE	FAH	p.P261L	Pathogenic	0.00015910(45/282876)	16.28	2.24 to 118.42	0.082722898
25	41	MALE	KIRC	WHITE	GJB2	p.KFIKG105-109X	Pathogenic	NA	NA	NA	NA
26	45	FEMALE	KIRC	WHITE	GJB2	p.Q57*	Pathogenic	0.00003187(9/282412)	81.29	10.27 to 643.20	3.73735E−05
27	47	FEMALE	KIRC	WHITE	MUTYH	p.R242H	Pathogenic	0.00008521(24/281670)	30.40	4.10 to 225.30	0.012249846
28	34	FEMALE	KIRC	BLACK OR AFRICAN AMERICAN	PMS2	p.LT731-732X	Pathogenic	NA	NA	NA	NA
29	29	MALE	KIRC	WHITE	TP53	p.H365Y	Pathogenic	NA	NA	NA	NA
30	23	FEMALE	KIRC	BLACK OR AFRICAN AMERICAN	VHL	p.C162F	Pathogenic	0.00000398(1/251440)	1306.18	118.19 to 14,435.42	1.3624E−106
31	38	FEMALE	KIRC	BLACK OR AFRICAN AMERICAN	VHL	p.C162F	Pathogenic	0.00000398(1/251440)	1306.18	118.19 to 14,435.42	1.3624E−106
32	50	FEMALE	KIRC	WHITE	VHL	p.R167W	Pathogenic	0.00000795(2/251448)	325.71	29.47 to 3599.59	5.93449E−13
33	29	FEMALE	KIRC	WHITE	XPC	p.V548X	Pathogenic	0.00002005(5/249358)	129.20	15.06 to 1108.48	5.01842E−07
34	33	MALE	KIRP	WHITE	ATM	p.R1875*	Pathogenic	0.00002002(5/249754)	173.44	20.20 to 1489.20	5.08026E−09
35	57	MALE	KIRP	WHITE	BRCA2	p.Q3066*	Pathogenic	NA	NA	NA	NA
36	47	MALE	KIRP	BLACK OR AFRICAN AMERICAN	FAH	p.G337S	Pathogenic	0.00007501(21/279946)	46.28	6.20 to 345.24	0.001643527
37	18	FEMALE	KIRP	BLACK OR AFRICAN AMERICAN	FH	p.S187*	Pathogenic	0.00000399(1/250648)	870.30	54.30 to 13,948.21	1.42543E−24
38	52	MALE	KIRP	WHITE	GJB2	p.E47*	Pathogenic	0.00000399(1/250772)	870.73	54.33 to 13,955.12	1.42543E−24
39	38	MALE	KIRP	WHITE	MET	p.H1112R	Pathogenic	0.00001203(3/249346)	871.83	175.22 to 4337.80	1.476E−195
40	35	MALE	KIRP	WHITE	MET	p.H1112R	Pathogenic	0.00001203(3/249346)	871.83	175.22 to 4337.80	1.476E−195
41	30	FEMALE	KIRP	WHITE	MET	p.H1112R	Pathogenic	0.00001203(3/249346)	871.83	175.22 to 4337.80	1.476E−195
42	37	MALE	KIRP	WHITE	MUTYH	p.E407GX	Pathogenic	0.00014170(40/282334)	49.18	11.83 to 204.46	3.90416E−12
43	57	MALE	KIRP	WHITE	MUTYH	p.E407GX	Pathogenic	0.00014170(40/282334)	49.18	11.83 to 204.46	3.90416E−12
44	62	MALE	KIRP	WHITE	PMS2	p.M1T	Pathogenic	0.00000400(1/250260)	868.95	54.22 to 13,926.62	1.48087E−24
45	43	MALE	KIRP	BLACK OR AFRICAN AMERICAN	RET	p.D631Y	Pathogenic	0.00000405(1/246932)	857.40	53.50 to 13,741.42	2.80654E−24
46	38	MALE	PRAD	[Unknown]	ATM	c.2921 + 1 N > A	Pathogenic	0.00002388(6/251304)	84.27	10.13 to 701.29	4.10188E−05
47	53	MALE	PRAD	WHITE	ATM	p.E1978*	Pathogenic	0.00004379(11/251182)	92.07	20.36 to 416.46	1.22898E−19
48	38	MALE	PRAD	[Unknown]	ATM	p.R3008C	Pathogenic	0.00001591(4/251428)	126.47	14.11 to 1133.57	1.08043E−06
49	51	MALE	PRAD	[Unknown]	ATM	p.R447*	Pathogenic	0.00000796(2/251290)	252.80	22.89 to 2792.61	2.47305E−10
50	49	MALE	PRAD	WHITE	BRCA1	p.Q1408*	Pathogenic	0.00000398(1/251400)	505.83	31.59 to 8098.72	6.97237E−15
51	38	MALE	PRAD	[Unknown]	BRCA1	p.R1772*	Pathogenic	0.00001414(4/282892)	142.30	15.88 to 1275.42	2.40779E−07
52	41	MALE	PRAD	[Unknown]	BRCA1	p.VN923-924X	Pathogenic	0.00000398(1/251024)	505.08	31.55 to 8086.61	7.07023E−15
53	40	MALE	PRAD	[Unknown]	BRIP1	p.V758VX	Pathogenic	0.00000796(2/251216)	252.73	22.88 to 2791.78	2.47305E−10
54	53	MALE	PRAD	[Unknown]	DKC1	p.S280R	Pathogenic	0.00027780(57/205151)	7.24	1.00 to 52.39	0.33671376
55	41	MALE	PRAD	[Unknown]	GALNT3	c.516-2A > T	Pathogenic	0.00003897(11/282266)	51.63	6.65 to 400.66	0.001068325
56	36	MALE	PRAD	[Unknown]	PALB2	p.P684X	Pathogenic	0.00000795(2/251460)	252.98	22.90 to 2794.49	2.47305E−10
57	44	MALE	PRAD	[Unknown]	PMS2	c.989-1G > T	Pathogenic	0.00000405(1/246764)	496.51	31.01 to 7949.38	1.18724E−14
58	41	MALE	PRAD	[Unknown]	RAD51C	p.R237*	Pathogenic	0.00001591(4/251374)	126.44	14.11 to 1133.32	1.08043E−06
59	44	MALE	PRAD	[Unknown]	SERPINA1	p.Y184*	Pathogenic	0.00017670(50/282896)	22.81	5.53 to 94.00	3.82838E−06
							Overall	0.00345284(938/271661)	14.88	11.80 to 18.77	2.2E−16

**Table 4:** Summary of patient and mutational status and OR for patients with likely pathogenic germline mutations in this study

Number	Age	Sex	Cancer type	Race	Gene	Protein change	Annotation	Allele Freqency in population	OR	95% CI	Adjusted P
1	63	MALE	BLCA	WHITE	ATM	n.93-2A > T	Likely pathogenic	0.00000399(1/250850)	610.34	38.11 to 9774.77	1.0243E−17
2	46	MALE	BLCA	WHITE	ATR	p.T1640RYX	Likely pathogenic	0.00000398(1/251370)	611.60	38.19 to 9795.04	9.9595E−18
3	66	MALE	BLCA	WHITE	BRIP1	p.K703NX	Likely pathogenic	0.00002125(6/282326)	114.48	13.75 to 953.08	1.5426E−06
4	60	MALE	BLCA	WHITE	CDKN2A	p.E74GX	Likely pathogenic	0.00001725(4/231826)	141.01	15.73 to 1264.36	2.6366E−07
5	48	MALE	BLCA	WHITE	CHEK2	c.444 + 1 N > A	Likely pathogenic	0.00000398(1/251274)	611.37	38.17 to 9791.29	9.9595E−18
6	36	MALE	BLCA	WHITE	COL7A1	p.Q783*	Likely pathogenic	NA	NA	NA	NA
7	53	MALE	BLCA	WHITE	DDB2	p.Q87*	Likely pathogenic	0.00000398(1/251216)	611.23	38.17 to 9789.03	9.9595E−18
8	49	MALE	BLCA	BLACK OR AFRICAN AMERICAN	EXT2	p.R498*	Likely pathogenic	0.00001415(4/282742)	171.98	19.18 to 1542.05	1.1832E−08
9	63	FEMALE	BLCA	WHITE	PRDM9	p.R77*	Likely pathogenic	0.00004407(11/249584)	55.20	7.11 to 428.57	0.00069828
10	55	MALE	BLCA	WHITE	RAD51C	p.T60X	Likely pathogenic	0.00001193(3/251400)	203.89	21.16 to 1964.25	1.7599E−09
11	31	MALE	BLCA	WHITE	RAD51C	p.Y75*	Likely pathogenic	0.00001193(3/251456)	203.94	21.17 to 1964.68	1.7599E−09
12	65	FEMALE	BLCA	ASIAN	RAD51D	p.E35*	Likely pathogenic	0.00003579(9/251448)	136.28	29.35 to 632.69	1.4611E−27
13	37	MALE	BLCA	ASIAN	RHBDF2	n.836-2A > T	Likely pathogenic	0.00001218(3/246374)	199.81	20.74 to 1924.98	2.5107E−09
14	50	MALE	KICH	WHITE	MSH6	p.E1359ELX	Likely pathogenic	NA	NA	NA	NA
15	55	MALE	KICH	WHITE	RECQL	p.556-?	Likely pathogenic	0.00027940(78/279214)	111.83	26.90 to 464.90	3.0482E−26
16	36	MALE	KIRC	WHITE	BAP1	c.375 + 1 N > A	Likely pathogenic	0.00000800(1/119830)	310.44	19.38 to 4972.32	1.2813E−09
17	36	FEMALE	KIRC	BLACK OR AFRICAN AMERICAN	BRIP1	p.M1244X	Likely pathogenic	0.00002484(7/281774)	104.28	12.80 to 849.62	3.7418E−06
18	35	FEMALE	KIRC	WHITE	CDKN1B	p.M1I	Likely pathogenic	0.00000806(2/248218)	321.52	29.09 to 3553.35	8.272E−13
19	61	MALE	KIRC	WHITE	COL7A1	p.L2219LX	Likely pathogenic	0.00000398(1/250976)	650.19	40.59 to 10,414.22	1.0707E−18
20	58	FEMALE	KIRC	WHITE	FANCM	p.-1740-1741X	Likely pathogenic	0.00000708(2/282464)	365.88	33.11 to 4043.60	2.1299E−14
21	54	MALE	KIRC	WHITE	POLE	p.S1827*	Likely pathogenic	NA	NA	NA	NA
22	54	FEMALE	KIRC	BLACK OR AFRICAN AMERICAN	POLE	p.W1624*	Likely pathogenic	0.00003186(1/31386)	81.31	5.08 to 1302.35	0.0023152
23	12	FEMALE	KIRC	WHITE	VHL	p.E134*	Likely pathogenic	NA	NA	NA	NA
24	30	MALE	KIRP	BLACK OR AFRICAN AMERICAN	BRCA1	p.-1237-1238X	Likely pathogenic	0.00000398(1/251278)	872.49	54.44 to 13,983.27	1.425E−24
25	39	MALE	KIRP	WHITE	EXT2	p.Y641*	Likely pathogenic	0.00003183(1/31412)	109.07	6.81 to 1748.02	0.00037813
26	33	MALE	KIRP	BLACK OR AFRICAN AMERICAN	FANCM	p.Q1844X	Likely pathogenic	0.00001193(3/251422)	290.99	30.18 to 2805.86	5.5171E−13
27	38	MALE	KIRP	WHITE	FANCM	p.Q600*	Likely pathogenic	NA	NA	NA	NA
28	18	FEMALE	KIRP	BLACK OR AFRICAN AMERICAN	FH	c.556-2A > T	Likely pathogenic	0.00000400(1/249830)	867.46	54.13 to 13,902.69	1.5558E−24
29	44	MALE	KIRP	WHITE	FH	p.M380NX	Likely pathogenic	0.00000398(1/251412)	872.95	54.47 to 13,990.73	1.425E−24
30	25	MALE	KIRP	Not avaiable	FH	p.NE361-362X	Likely pathogenic	0.00000398(1/251108)	871.90	54.40 to 13,973.81	1.425E−24
31	54	MALE	KIRP	WHITE	GJB2	p.Y65*	Likely pathogenic	NA	NA	NA	NA
32	53	MALE	KIRP	WHITE	MAX	p.Y71*	Likely pathogenic	0.00000398(1/251312)	872.61	54.45 to 13,985.17	1.425E−24
33	42	FEMALE	KIRP	WHITE	MSH2	p.Q915*	Likely pathogenic	0.00000844(2/236964)	411.39	37.20 to 4549.75	5.3559E−16
34	21	MALE	KIRP	WHITE	PMS2	p.-371-372X	Likely pathogenic	NA	NA	NA	NA
35	28	FEMALE	KIRP	WHITE	POT1	p.R363*	Likely pathogenic	0.00002500(7/280046)	278.78	57.67 to 1347.75	4.1526E−53
36	47	MALE	KIRP	BLACK OR AFRICAN AMERICAN	PRKAR1A	p.Y138*	Likely pathogenic	0.00000398(1/251326)	872.66	54.45 to 13,985.94	1.425E−24
37	52	MALE	KIRP	Not avaiable	RECQL	p.556-?	Likely pathogenic	0.00027940(78/279214)	24.94	6.10 to 101.98	1.0827E−06
38	35	MALE	PRAD	Not Avaiable	ATM	p.E1267X	Likely pathogenic	NA	NA	NA	NA
39	48	MALE	PRAD	WHITE	ATM	p.L263X	Likely pathogenic	NA	NA	NA	NA
40	38	MALE	PRAD	BLACK OR AFRICAN AMERICAN	ATM	p.LT2332-2333X	Likely pathogenic	NA	NA	NA	NA
41	52	MALE	PRAD	Not avaiable	BRCA2	p.A1564AX	Likely pathogenic	NA	NA	NA	NA
42	46	MALE	PRAD	WHITE	EPCAM	c.426-1 N > C	Likely pathogenic	NA	NA	NA	NA
43	31	MALE	PRAD	WHITE	MSH2	p.F136X	Likely pathogenic	0.00000398(1/251332)	505.70	31.58 to 8096.53	6.9724E−15
44	50	MALE	PRAD	Not avaiable	MTAP	p.F78X	Likely pathogenic	0.00000442(1/226276)	455.28	28.44 to 7289.37	1.5451E−13
45	39	MALE	PRAD	Not avaiable	PMS2	p.669-?	Likely pathogenic	NA	NA	NA	NA
46	36	MALE	PRAD	Not avaiable	PMS2	p.R563*	Likely pathogenic	0.00000796(2/251330)	252.85	22.89 to 2793.05	2.4731E−10
47	40	MALE	PRAD	Not avaiable	POT1	p.R363*	Likely pathogenic	0.00002500(7/280046)	161.31	33.43 to 778.45	5.4259E−31
48	30	MALE	PRAD	Not avaiable	RAD51D	p.E35*	Likely pathogenic	0.00003579(9/251448)	112.65	24.28 to 522.71	3.8539E−23
49	44	MALE	PRAD	WHITE	RECQL	p.KN487-488X	Likely pathogenic	NA	NA	NA	NA
50	34	MALE	PRAD	WHITE	TSC1	p.Q550*	Likely pathogenic	0.00002123(6/282572)	94.76	11.39 to 788.54	1.2966E−05
51	41	MALE	PRAD	Not avaiable	TSC2	p.M1V	Likely pathogenic	0.00005311(15/282430)	37.88	4.99 to 287.35	0.00510767
52	36	MALE	PRAD	Not avaiable	UROD	p.R365*	Likely pathogenic	0.00001591(4/251480)	126.50	14.11 to 1133.80	1.0804E−06
							Overall	0.00109559(281/256482)	33.18	24.90 to 44.20	2.2E−16

## Discussion

In this study, we systematically investigated the characteristics of germline mutations and relevant phenotypes in five types of genitourinary cancers, and found a series of features, including highly cancer type-dependent top mutated genes, predominant mutation types with large fragment alterations, male-dominant patient distribution and age-irrelevant cancer onset. We also revealed significant correlation between pathogenic/likely pathogenic mutations and patient prognosis and the risk of genitourinary cancers in population, suggesting them as prognostic and risk factors. Our study established the clinical relevance of these mutations and highlighted the importance of early detection and intervention in population with pathogenic and likely pathogenic germline mutations.

Cancer patients with pathogenic or likely pathogenic germline mutations are a special group of patients with characteristic phenotypes, including early onset cancers, familial aggregation, multiple organ involvement, high level of malignancy, poor therapeutic response and poor prognosis [13–16]. The most commonly seen cancers with definite causes of germline mutations include Lynch syndrome and hereditary breast and ovarian cancer (HBOC) [17, 18], while recent evidence suggested that a subset patients with pathogenic germline mutations were also predisposed to higher lung cancer risk and familial aggregation [19–21]. It was reported that genes responsible for DNA damage repair (DDR) were mainly involved in germline mutations in hereditary cancers [8, 22]. This includes a series of genes, such as *MLH1*, *MSH2*, *MSH6*, *PMS2*, *ATM*, *BLM*, *BRCA1*, *BRCA2*, *POLE* and *POLD1* [8, 22, 23]. Germline mutations of these genes may greatly enhance the risk of cancer, and certain group of mutations may correspond to certain cancer types. For example, genes in mismatch repair (MMR) (*MLH1*, *MSH2*, *MSH6*, *PMS2*, etc.) are predominantly linked to Lynch syndrome, and genes in homologous recombination repair (HRR) (*BRCA1* and *BRCA2*) are mainly involved in HBOC. Other less-frequent germline mutations are less cancer type-specific and may be found in any cancer.

Although hereditary cancers such as Lynch syndrome and HBOC have been widely studied, the germline mutational status in genitourinary cancers and their correlation with prognosis and cancer risk have been largely uninvestigated. This is possibly due to the low incidence of germline mutation-induced genitourinary cancer and the fact that there have been few definite links between certain germline mutations and certain type of genitourinary cancer [24]. We therefore performed a database research and revealed interesting characteristics of germline mutations in genitourinary cancer and established their correlation with patient prognosis. It was not surprising to find that *ATM*, *BRCA1*, *PMS2* and *BRCA2* were among genes with highest number of mutations. As mentioned above, these genes belong to DDR and are sensitive to DNA damage. DNA damage is a common process happened during carcinogenesis, and factors including chronic inflammation, virus infection, carcinogen or toxin can all lead to DNA damage which initiate repair [25–27]. In normal tissues of subjects without germline mutations, repetitive damage and repair alter the microenvironment and the normal cellular cycle controlled by a series of epigenetic and genetic mechanisms. Abnormal gene regulation under repetitive damage and repair ultimately leads to accumulation of somatic mutations, and key mutations at driver genes result in malignant transformation of cells [28–30]. In contrast, for subjects with germline mutations at DDR genes, the DNA repair mechanism is impaired congenitally, cellular malignant transformation may therefore happen at early stage of life and lead to tumor growth. This is the reason for high cancer incidence and low onset age for people with Lynch syndrome or HBOC-related germline mutations [25–27].

*ATM* gene mutations were also reported in recent studies on germline mutations in non-small cell lung cancer (NSCLC) [21, 31, 32]. It was reported to be the gene with highest germline mutation frequency in western population [32]. Similarly, *BRCA1*, *BRCA2* and *PMS2* were also reported as germline mutations in cancers other than Lynch syndrome and HBOC [21, 31, 32]. *BLM* and *VHL* genes also contained germline mutations leading to Bloom syndrome [33] and Von Hippel-Lindau (VHL) disease [34], respectively. However, they were also found in other cancers with germline mutations. It is possible that DDR gene germline mutations can cause various types of cancers, with MMR genes preferentially found in Lynch syndrome and HRR genes preferentially found in HBOC. Functional subgroups of DDR genes may differentially affect carcinogenesis of different tissues.

It appeared from our study that bladder cancer and prostate cancer shared some common top mutated genes, while the top mutated genes were quite different in kidney cancer. In kidney cancer, two KIRC and one KIRP patients carried *GJB2*, three KIRP patients carried *MET*, one KIRC and two KRIP patients carried *MUTYH* and three KIRC patients carried *VHL* germline mutations. GJB2 germline mutations have been previously reported in congenital hearing loss [35] and rarely been reported in cancer [36]. Our study revealed *GJB2* germline pathogenic mutations in KIRC and KIRP for the first time, providing new evidence for the pathogenicity of the gene in kidney cancers. In contrast, *MET* germline mutations have been implicated in many cancers, including KRIP [37], and it was not surprising that we also found MET germline mutations in this study. Similarly, *MUTYH* germline mutations have also been reported in many cancers, including kidney cancer [38, 39]. The germline *VHL* mutations have been linked to VHL disease, which is an inheritable condition leading to retinal and central nervous system hemangioblastomas, clear cell renal cell carcinomas, pheochromocytomas, pancreatic neuroendocrine tumors and endolymphatic sac tumors [40]. From our observations,.these top mutated genes were kidney-specific and distinguish themselves from those in bladder and prostate cancers, although they all belong to genitourinary cancer. Therefore, the mechanism of aberrancies in kidney cancers with germline mutations may be largely different from that of bladder and prostate cancer.

Determination of pathogenicity of germline mutations is crucial for establishing the link between mutations and phenotypes. Here in this study we interpreted the pathogenicity of all reported mutations based on ACMG guidelines. Frameshift and stop gained mutations were highly possible pathogenic or likely pathogenic mutations, indicating the inherit property of mutations. The interpretation would be more meaningful if the pathogenic mutations happened to key DDR genes related to known phenotypes. In contrast, missense mutations are more difficult to interpret, unless sufficient evidence is available to link single amino acid change with phenotypes. This is more likely to occur in single-gene related hereditary diseases, such as VHL disease [34, 41, 42]. In our study, pathogenic mutations of *VHL* gene were all missense mutations, reflecting the intrinsic properties of mutations in this disease. In contrast, the ratio of missense mutations was low in other highly mutated genes, such as *ATM*, *BRCA1* and *PMS2*. It was interesting to find that nearly half of the mutations found in BLCA were stop gained mutations. These mutations spread many genes including both high and low frequency genes. This observation demonstrated characteristic mutational change in BLCA, suggesting high ratio of truncated DDR related proteins in the specific cancer.

It is widely known that male overweight female in patient number with a rough ratio of 2:1 in sporadic kidney and urothelial cancer [43]. We found similar trend in genitourinary cancer with germline mutations, suggesting that male is possibly more susceptible to genitourinary cancer if the chance of mutation heredity is similar for both sexes. It is also possible that male may have higher penetrance than female. KIRC is the most common type of kidney cancer, and it was interesting that female overweight male in the number of KIRC patients with germline mutations, although male overweight female in sporadic KIRC [43]. The reason for this discrepancy may include the manner of heredity, mutation penetrance and environmental factors. Previous studies on Lynch syndrome and HBOC revealed significantly lower onset age compared with sporadic colorectal, breast and ovarian cancer patients [44, 45]. However, we did not find age difference between those with pathogenic or likely pathogenic mutations and those with non-pathogenic mutations. Since non-pathogenic mutations were comprehensive found in sporadic cancer patients and normal subjects, our observation suggested that germline mutations of genitourinary cancers did not affect the onset age.

Previous studies reported that untreated patients with Lynch syndrome or HBOC exhibited significantly worse prognosis than sporadic patients [13–16]. Our study with genitourinary cancer patients also showed identical trend, in which patients with pathogenic or likely pathogenic mutations exhibited much worse overall survival rate than those with non-pathogenic mutations, suggesting that pathogenic and likely pathogenic germline mutations were risk factors for the prognosis of genitourinary cancer patients. Furthermore, the OR values we calculated strongly supported the notion that those with pathogenic or likely pathogenic germline mutations were in much higher risk for developing all types of genitourinary cancers than those without the germline mutations (general population). Our observation of higher overall OR in likely pathogenic mutations than pathogenic mutations suggested that some likely pathogenic mutation may be essentially pathogenic, although limited available evidence does not support the pathogenic interpretation currently. These observations provided strong evidence for the necessity of early detection of germline mutations for those with strong family history and continuous surveillance and early intervention for those with confirmed pathogenic or likely pathogenic germline mutations. On the other hand, it appeared from our study that no difference was found in overall survival rate between patients with pathogenic mutations and those with likely pathogenic mutations. This suggested that some likely pathogenic mutations may actually be pathogenic, although clinical evidence may be absent for interpretation of pathogenic for many likely pathogenic mutations, especially for frameshift and stop gained mutations.

Genitourinary cancer patients with pathogenic or likely pathogenic mutations may be treated with corresponding targeted drugs based on the availability of matched drugs for certain germline mutations. For example, locally advanced or metastatic genitourinary cancer patients with *BRCA1/2* mutations may be treated with poly ADP-ribose polymerase (PARP) inhibitors. Although it has long been known that PARP inhibitors were effective for prostate cancer with pathogenic or likely pathogenic *BRCA1/2* mutations [46, 47], it was not until recently that evidence started to emerge that PARP inhibitors were also effective in other genitourinary malignancies [47–51], except in renal cell carcinoma, since no evidence on the presence of *BRCA1/2* mutations has been available in the cancer [52]. Similarly, locally advanced or metastatic cancer patients with germline mutations of MMR genes may be treated with immune checkpoint inhibitors such as PD-l inhibitors, as these cancers generally exhibit high tumor mutational burden and/or high microsatellite instability [53]. Future development of targeted drugs for DDR pathway may open the door for new treatment strategies for genitourinary cancer patients with germline mutations.

This study had some limitations. First, the number of genitourinary cancer patients was limited since data was available from only 206 patients, which led to the limited number of patients in each individual cancer. Secondly, due to the lack of ethnic diversity and predominant male population, the current findings could be non-generalizable to non-White and female patients. Thirdly, the prognosis of patients may be influenced by therapeutic strategies, however, the information of therapy is not available in the TCGA database.

## Conclusions

In this study, the germline mutational characteristics for genitourinary cancers have been comprehensively investigated. A series of pathogenic and likely pathogenic germline mutations have been defined and their mutational landscape in several genitourinary cancers has been studied. Pathogenic and likely pathogenic germline mutations increased the risk and indicated poor prognosis of genitourinary cancers.

## Data Availability

Data are available for download by visiting the following website, and can be downloaded through the link of supplementary information: https://www.sciencedirect.com/science/article/pii/S0092867418303635. Alternatively, data can be downloaded directly from the link below: https://ars.els-cdn.com/content/image/1-s2.0-S0092867418303635-mmc2.xlsx. For original dataset source of the germline mutations, the raw data can be accessed at ISB cancer genome cloud (ISB-CGC) at the link below for qualified applicants: http://isb-cancer-genomics-cloud.readthedocs.io/en/latest/sections/webapp/Gaining-Access-To-Contolled-Access-Data.html.
